# Combined spinal–epidural anesthesia for radical hysterectomy in a patient with Sjȍgren syndrome with progressive interstitial lung disease

**DOI:** 10.1186/s40064-016-3352-5

**Published:** 2016-10-06

**Authors:** Jeong-Min Hong, Eunsoo Kim, Hae-Kyu Kim, Do-Won Lee, Ji-Seok Baik, Ji-Youn Lee

**Affiliations:** 1 Department of Anesthesia and Pain Medicine, School of Medicine, Pusan National University, Busan, Korea; 2 Biomed Research Institute, Pusan National University Hospital, Busan, Korea

**Keywords:** Combined spinal–epidural anesthesia, Interstitial lung disease, Radical hysterectomy, Sjȍgren syndrome

## Abstract

**Introduction:**

Interstitial lung disease (ILD), which is the most common form of respiratory involvement of Sjȍgren syndrome (SS), is highly associated with postoperative pulmonary complications after surgery. We report the successful anesthetic management of a cervical cancer patient with SS and ILD under combined spinal-epidural anesthesia (CSE) to avoid postoperative pulmonary complications.

**Case description:**

A 41-year-old woman with SS complicated by recently progressive ILD was scheduled for an elective radical hysterectomy under the diagnosis of cervical cancer. We performed CSE with separate needle technique (SNT) using two different interspaces. An epidural catheter was inserted at T11–T12 before administration of spinal medication at L3–L4. We could achieve successful anesthetic management for radical hysterectomy, maintaining stable hemodynamic variables. Postoperative analgesia, using epidural catheter, was effective and devoid of any postoperative pulmonary morbidity.

**Discussion and Evaluation:**

CSE could offer a high level of sensory blockade, profound muscular blockade, longer duration of surgical anesthesia, excellent postoperative pain control, and reduction in the incidence of pulmonary morbidity. Therefore it would be excellent anesthetic option for the patients with pulmonary impairment.

**Conclusion:**

CSE with SNT may be particularly advantageous in patients with pulmonary impairment such as progressive ILD when general anesthesia is associated with high risk of postoperative complications.

## Background

Sjȍgren syndrome (SS) is a chronic inflammatory autoimmune disease characterized by lymphocytic infiltration of exocrine glands resulting in sicca symptoms, usually xerostomia and dry eyes (Kokosi et al. 2010). It may also have extragrandular involvement including lung, thyroid, kidney, and liver. Pulmonary involvement has been frequently reported in SS (Strimlan et al. 1976; Constantopoulos et al. 1985). Diffuse interstitial lung disease (ILD) is the most common form of respiratory involvement in SS (Kokosi et al. 2010). The patients with ILD may have a higher incidence of postoperative pulmonary complications after surgery (Choi et al. 2014). We report the successful anesthetic management of a cervical cancer patient with SS and ILD under combined spinal–epidural anesthesia.

## Case presentation

**Fig. 1:**
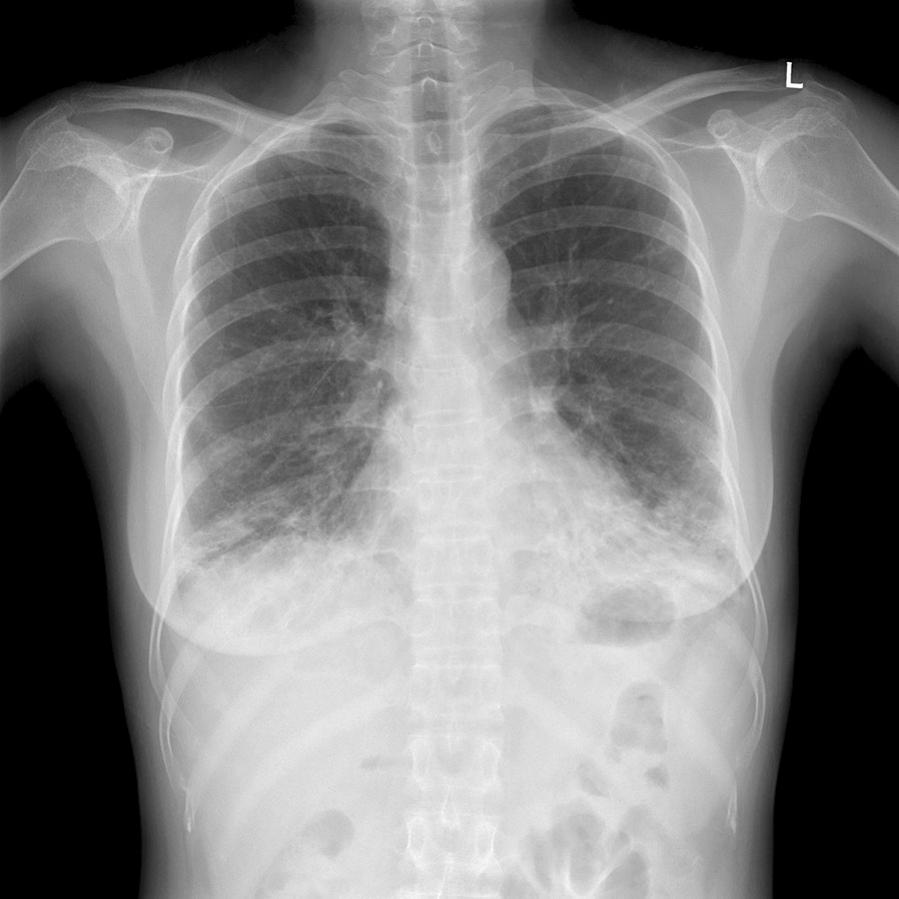
Preoperative chest radiograph. A chest radiograph showed an increased reticular opacity at both lower lung zone in preoperative evaluation

**Fig. 2:**
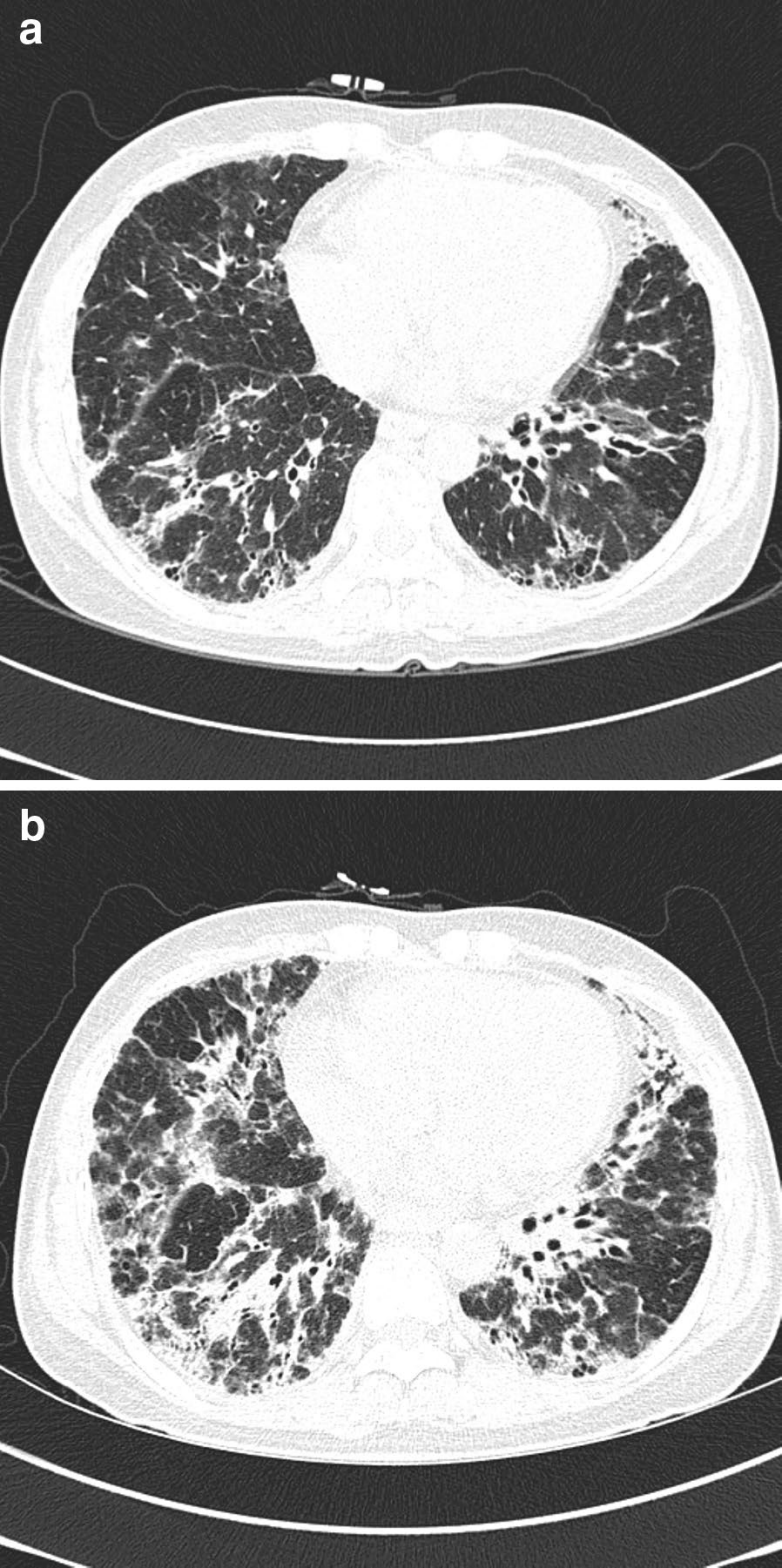
High-resolution computed tomography (HRCT) of lung. **a** HRCT (1 year ago) showed traction bronchiectasis and consolidation in bilateral subpleural and lower lung zone. **b** Recent HRCT showed an increased extent of coarse reticulation, ground glass opacity with traction bronchiectasis in bilateral subpleural and lower lung zone. Increased extent of coarse reticulation, ground glass opacity with traction bronchiectasis in bilateral subpleural and lower lung zone

A 41-year-old woman (height, 162.2 cm; weight, 50.4 kg) with a 4-year history of SS was scheduled for an elective radical hysterectomy with bilateral pelvic lymph node dissection and ovary transposition under the diagnosis of cervical cancer. She had been diagnosed with SS accidentally on a preoperative evaluation when she sought treatment for a radius fracture 4 years ago. She presented symptoms of dyspnea, dry mouth, dry eyes, Raynaud’s syndrome and arthralgia of the hand and knee. On preoperative evaluation, chest X-ray (CXR) showed increased reticular opacity in both lower lung zones, and high-resolution computed tomography (HRCT) demonstrated increased extent of coarse reticulation, and ground glass opacity with traction bronchiectasis in bilateral subpleural and lower lung zone corresponding to fibrotic nonspecific interstitial pneumonia pattern compared to previous HRCT (Figs. 1, 2). Pulmonary function test (PFT) was also aggravated. Current PFT indicated a moderate restrictive pattern with negative effect of bronchodilator test (FVC 1.97 L,  %FVC 64 %, FEV_1_ 1.74 L, FEV_1_/FVC 88 %) while previous PFT indicated a mild restrictive pattern (FVC 2.29 L,  %FVC 56 %, FEV_1_ 1.92 L, FEV_1_/FVC 84 %). Arterial blood gas analysis (ABGA) revealed pH 7.419, PCO_2_ 36.9 mmHg, PO_2_ 92.1 mmHg, and oxygen saturation at 96.1 %. Thus, in comparison with previous result of HRCT and PFT, ILD associated with SS was showing recent worsening. The rheumatologist recommended a high dose steroid therapy to prevent disease progression and suggested that her operation be postponed for 2 months, considering the stage of the disease. However, it did not guarantee the improvement of lung function.

After consideration of the potential risks and benefits, we decided to perform combined spinal–epidural anesthesia (CSE) for the surgery. However, we also had a plan to postpone surgery if CSE technique failed. The patient arrived with graduated compression stockings in the operating room. Before induction of anesthesia, a routine monitoring was established and hydrocortisone 100 mg was administered intravenously. Using 2–5 MHz curved array probe connected to ultrasound (S-nerve, Sonosite Inc, Bothell, WA, USA), measurement of the depth of the epidural space and confirmation of interlaminar space was performed with paramedian sagittal oblique and transverse interlaminar view for a successful block. An epidural catheter was placed through the T11–12 intervertebral space before the subarachnoid block, checked by the aspiration test and by administering an epidural test dose (3 ml of 2 % lidocaine with epinephrine 15 µg) to rule out its inadvertent intrathecal or intravascular placement. Subsequently, a 25-gauge spinal needle was inserted at the L3-4 intervertebral space and 0.5 % hyperbaric bupivacaine 15 mg with fentanyl 25 µg was injected into the subarachnoid space. The sensory blockade to T4 dermatome, was achieved 10 min after spinal medication, as assessed by the pinprick method in the supine position. Surgical anesthesia for radical hysterectomy was achieved without epidural injection. One hour later, 10 ml of 0.75 % ropivacaine with fentanyl 75 µg was injected into the epidural space. Dexmedetomidine infusion was started at 1 mg/kg loading dose over 10 min and then 0.2 to 1.0 µg/kg/h of maintenance infusion titrated to level of sedation and hemodynamic variables was continued. Supplemental oxygen (2 to 3 L/min) was provided, because SpO_2_ had decreased to 93 % after achieving higher spinal segment blockade. ABGA monitoring was performed repeatedly throughout the surgery (Table 1). The patient did not complain of pain during the surgery and her vital signs were stable without any vasoactive drugs except the SpO_2_ had decreased to 90 % when she was fully sedated. The surgical procedure was performed to completion uneventfully. The duration of the operation was 135 min. Estimated blood loss was 400 ml and her urine output was about 120 ml; 2400 ml of crystalloid solution was administered.

**Table 1:** Serial arterial blood gas measurements during surgery

	pH	PCO_2_ (mmHg)	PO_2_ (mmHg)	SaO_2_ (%)	Nasal O_2_ supplement (L/min)
After CSE	7.337	32.7	71	93	2
After sedation	7.334	33.4	61	90	3
After main procedure	7.290	37.4	87	95	3
Discharge from PACU	7.353	37.6	93	97	3

*CSE* combined spinal–epidural analgesia, *PACU* postanesthesia care unit

Postoperatively, patient controlled epidural analgesia (PCEA) was provided with PCEA pump using 0.2 % ropivacaine with 3 µg/ml fentanyl (basal infusion 2 ml/h, demand dose 3 ml, lock-out interval 30 min, total volume 150 ml). The numeric rating scale was 0–2 while using PCEA without any rescue medication, and the patient was satisfied with pain management. The patient was discharged without any postoperative pulmonary complications on postoperative day 10.

## Discussion

This case report describes the successful use of CSE in a patient with SS and aggregation of ILD for radical hysterectomy with BPLD. SS can be classified as primary or secondary associated with other connective tissue disease such as rheumatoid arthritis, systemic lupus erythematosus. Most commonly respiratory system is involved among extragrandular organs in SS and respiratory involvement has been reported in 9–75 % (Kokosi et al. 2010; Strimlan et al. 1976; Constantopoulos et al. 1985). Pulmonary manifestation in SS is mainly associated with ILD including nonspecific interstitial pneumonia, lymphocytic interstitial pneumonia, usual interstitial pneumonia, and organizing pneumonia (Kokosi et al. 2010). Postoperative pulmonary complications (PPCs) are an important cause of postoperative morbidity, mortality. PPCs included pneumonia, atelectasis, respiratory failure, and exacerbation of underlying chronic lung disease. A population-based surgical cohort study reported a 5 % incidence of PPCs across all types of surgery (Canet et al. 2010). But, the incidence of PPCs in a patient with ILD is higher than that reported in a broad surgical population (11 vs. 5 %) (Choi et al. 2014). Patients with ILD had a higher incidence of postoperative acute respiratory distress syndrome (13 vs. 1.8 %) and higher postoperative mortality (8 vs. 1.4 %, P < 0.01) than those without ILD in lung surgery (Voltolini et al. 2013). In our case, the patient had ILD associated with SS, with recent worsening of respiratory function. Normally, radical hysterectomy and pelvic lymph node dissection for cervical cancer is performed under general anesthesia. However, there was a high possibility of PPCs if our patient underwent the surgery under general anesthesia. And, regional anesthesia can improve diaphragmatic function by disrupting surgery-induced reflex inhibition of the phrenic nerve and increase chest wall compliance and avoid operative endotracheal intubation and mechanical ventilation. Thus, we decided to perform CSE because it would offer a high level of sensory blockade, profound muscular blockade, longer duration of surgical anesthesia, excellent postoperative pain control, and reduction in the incidence of pulmonary morbidity (Jayanthi et al. 2007; Ballantyne et al. 1998; Guay et al. 2014). Avoidance of fluid overload seems to be important factor to prevent PPCs. The administration of intravenous crystalloid can help to minimize decrease of blood pressure caused by blockade of the peripheral and cardiac sympathetic fibers after neuroaxial block. However, PPCs associated with pulmonary edema could be caused and exacerbated by fluid overload. Therefore, it may be preferable to support blood pressure with vasopressors in this circumstance.

Recently, the application of ultrasound has been growing in regional anesthesia and pain practice. Despite the limitations of ultrasound imaging of the spine, there are current evidences for its clinical utility in ultrasound-assisted neuraxial blockade. Neuraxial ultrasound can improve the success rate of block by providing anatomical information and decreasing complications by reducing the number of needle manipulations for the puncture (Perlas et al. 2014; Grau et al. 2004). It was not necessary for this patient to apply ultrasound because she was thin and had easily palpable bony landmarks of the spine. However, the pre-procedural ultrasound examination of spine was done to provide localization of epidural space and its preceding structure for a successful block and to facilitate CSE at intended level of spine for effective surgical anesthesia.

CSE has good characteristics of both spinal and epidural anesthesia and is devoid of their respective drawbacks. It facilitates a rapid onset, profound and prolonged block along with an effective postoperative pain control. Various CSE techniques have been introduced and modified to improve the success rate and decrease the complications. Needle-through-needle technique (NTN) and separate needle technique (SNT) are commonly used in clinical practice. SNT may be performed with spinal blockade and epidural catheter placement either at the same or two different interlaminar spaces. Some studies have reported a lower failure rate in SNT using double space compared with NTN (Lyons et al. 1992; Backe et al. 2004). It may decrease the risk of complications like inadvertent intravascular or intrathecal catheter migration if proper placement of epidural catheter can be tested before subarachnoid blockade (Jayanthi et al. 2007). However, SNT may need a longer time to perform and thus cause more discomfort during the procedure than NTN (Callesen et al. 1999). In this case, CSE with SNT at two interspaces (T11/12 for epidural catheter placement, L3/4 for spinal anesthesia) was performed to achieve higher success rate and to avoid complications. There was very little chance of epidural catheter damage, because needles insertion site was too far and epidural catheter was placed upward through the T11–12 intervertebral space.

Dexmedetomidine is a centrally acting α-2 adrenoceptor agonist that can provide sedation without risk of respiratory depression. In addition, intravenous dexmedetomidine can prolong the duration of sensory and motor blocks as well as the time to first analgesic request of spinal anesthesia (Abdallah et al. 2013). This is the reason why dexmedetomidne is used as a sedative agent in our patient.

## Conclusion

We report the successful use of CSE in a patient with SS and aggravated ILD undergoing radical hysterectomy. Ultrasound-assisted CSE may be particularly advantageous in patients with coexisting pulmonary impairment such as progressive ILD, while general anesthesia is associated with high possibility of PPCs compared to those associated with regional anesthesia.

## Abbreviations

SS: Sjȍgren syndrome
ILD: interstitial lung disease
CSE: combined spinal–epidural anesthesia
SNT: separate needle technique
CXR: chest X-ray
HRCT: high-resolution computed tomography
PFT: pulmonary function test
ABGA: arterial blood gas analysis
PCEA: patient controlled epidural analgesia
PPCs: postoperative pulmonary complications
NTN: needle-through-needle technique
SNT: separate needle technique
